# Human neutrophils require short exposure to cytokines and allergen to become functional antigen‐presenting cells

**DOI:** 10.1111/all.15460

**Published:** 2022-08-06

**Authors:** Dominika Polak, Adelheid Elbe‐Bürger, Claudia Kitzmüller, Gerhard J. Zlabinger, Barbara Bohle

**Affiliations:** ^1^ Institute of Pathophysiology and Allergy Research, Center for Pathophysiology, Infectiology and Immunology Medical University of Vienna Vienna Austria; ^2^ Department of Dermatology Medical University of Vienna Vienna Austria; ^3^ Institute of Immunology Medical University of Vienna Vienna Austria

**Abbreviations**
AIT: allergen‐specific immunotherapy
APC: antigen‐presenting cell
cpm: counts per minute
dpm: delta cpm
GM‐CSF: granulocyte macrophage colony‐stimulating factor
IFN: interferon
IL: interleukin
LPR: late phase reaction
PBMC: peripheral blood mononuclear cells
SI: stimulation index
TCL: T‐cell line
TNF: tumor necrosis factor

To the Editor,

High numbers of neutrophils accumulate in late‐phase reactions (LPRs) of IgE‐mediated allergy. We previously reported that neutrophils exposed to GM–CSF (granulocyte macrophage colony‐stimulating factor), IL‐3 (interleukin 3), and IFN‐γ (interferon) for 18–24 h can present the prototypic major birch pollen allergen Bet v 1 to specific CD4^+^ T‐cells, triggering their proliferation and cytokine production.^1^ The ex vivo detection of Human leukocyte antigen–DR (HLA–DR)‐positive neutrophils in cutaneous allergic LPR^1^ together with the neutrophil‐induced aggravation of allergen‐induced T‐cell‐mediated airway hyperresponsiveness in a humanized mouse model^2^ tempted us to propose that neutrophils amplify allergic inflammation also by acting as antigen‐presenting cell (APC). As the information on the timeline of this contribution is scarce, we aimed to study the periods of cytokine and allergen exposure essential for neutrophils to become APC by a sophisticated approach involving Bet v 1‐specific T‐cell lines (TCL) and autologous >99% pure neutrophils from birch pollen‐allergic individuals (for details, see Appendix S1).

Neutrophils were incubated with GM–CSF, IL‐3, and IFN‐γ for 1–12 h and after 18–24 h added to TCL without and with Bet v 1 (Figure 1). Cytokine exposure of only 1 h converted neutrophils into APC inducing allergen‐specific proliferation of 7/8 TCL. After 3 h of cytokine‐exposure, all TCL responded. In parallel, we observed that neutrophils displayed maximum HLA–DR expression after 1 h and significantly prolonged viability after 3 h of cytokine exposure (Figure S1). These results indicated that very few hours in the cytokine milieu of allergic LPR suffice to transform neutrophils into functional APC. We confirmed that sensitized primary skin mast cells produce GM‐CSF and low amounts of IL‐3 and IFN‐γ following IgE‐mediated degranulation (Table S1). As GM–CSF was the most prominent cytokine released by the mast cells and previously determined as a potent trigger of neutrophilic antigen‐presenting activity,^3^ we analyzed its timeline of action on neutrophils. Again, only 1 h of exposure transformed neutrophils into functional APC (Figure 1B). Earlier work has demonstrated that GM–CSF is measurable 4–6 h after IgE‐mediated degranulation of mast cells and peaks by 12 h.^4^ Furthermore, TNF‐α was described to be released within minutes from intracellular stores of mast cells followed by de novo synthesis thereafter.^5^ Similar to GM–CSF, TNF‐α promoted allergen‐uptake and HLA–DR expression of neutrophils^3^ and was found in the supernatants of IgE‐degranulated mast cells (Table S1). Neutrophils appear in LPR at around 4 h following allergen stimulation.^6^ Therefore, it is conceivable that they encounter these cytokines on their arrival and after 1 h, begin their conversion in APC, which is finished on the arrival of allergen‐specific T‐cells at 18–24 h after the mast cell degranulation.^6^ As it is very speculative to extrapolate the in vitro properties of single cytokines to the situation in vivo, we exposed neutrophils to the complex mixture of factors released from autologous allergen‐activated basophils (for details, see Appendix S1). Supernatants of Bet v 1‐stimulated but not of unstimulated basophils induced HLA–DR expression on neutrophils (Figure S2).

**FIGURE 1:**
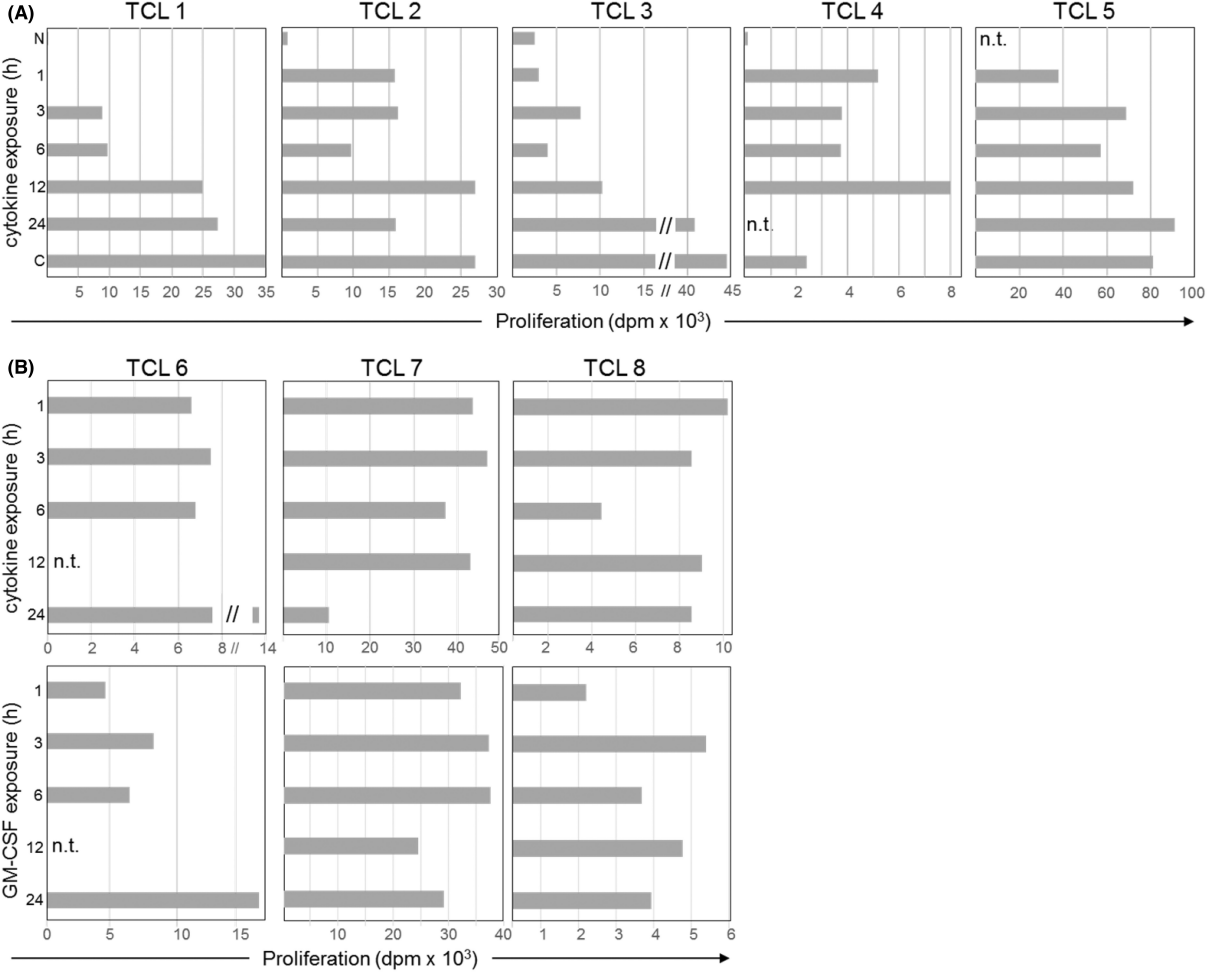
Cytokine exposure period and T‐cell‐stimulatory capacity of neutrophils. (A) Neutrophils were incubated with GM–CSF, IL‐3, and IFN‐γ for the indicated period, washed, maintained in medium for 18–24 h, and added to Bet v 1‐specific TCL without and with Bet v 1. T‐cell proliferation was measured after 48 h, dpm = cpm of allergen‐stimulated cultures minus cpm of medium controls; C, neutrophils exposed to cytokines without washing; N, neutrophils not exposed to cytokines. (B) Neutrophils were incubated with GM‐CSF, IL‐3, and IFN‐γ or GM–CSF alone (lower panel).

Finally, we studied the period of allergen exposure required for T‐cell activation and incubated neutrophils for 1–24 h with Bet v 1 before addition to specific TCL (Figure 2). Considering a SI >2 as positive, 2/3 TCL proliferated after a pulsing period of 3 h. All the TCL proliferated after allergen pulsing for 6 h. These results matched our finding that neutrophils rapidly process Bet v 1 into fragments encompassing relevant T‐cell epitopes.^1^ Altogether, the here proposed chronology of neutrophilic action as APC makes their contribution to allergic inflammation plausible. Certainly, the situation in situ is more complex including parameters not considered by this study. Still, our results obtained with pure neutrophils, a single prototypic allergen, autologous allergen‐specific T‐cells and effector cells support that the HLA–DR‐positive neutrophils detected in allergic LPR 20–24 h after allergen injection represent functional APC.^1^

**FIGURE 2:**
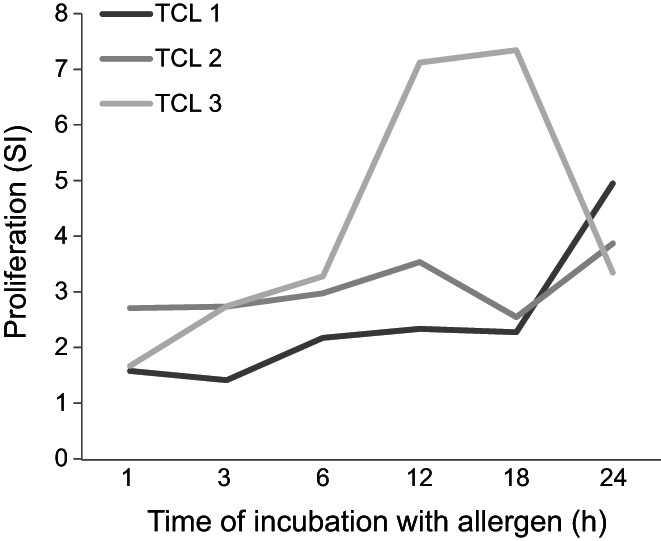
Allergen exposure period and antigen‐presenting capacity of neutrophils. Neutrophils were incubated for indicated periods without or with allergen within a total period of 24 h in the presence of cytokines and added to autologous allergen‐specific TCL. T‐cell proliferation was measured after 48 h, SI = cpm of stimulated cultures/cpm of medium controls.

## AUTHORS CONTRIBUTIONS

DP and BB designed the experiments; DP and CK performed the experiments; AEB provided human skin mast cells; GJZ provided cytokine measurements; and DP and BB analyzed the data and wrote the manuscript.

## CONFLICT OF INTEREST

The authors have nothing to disclose.

## Supporting information


Appendix S1
