# Supplementary material for: Human neutrophils require short exposure to cytokines and allergen to become functional antigen‐presenting cells
Source: Allergy. 2022 Aug 6;78(1):291–3. doi: 10.1111/all.15460 (PMC10087710; doi:10.1111/all.15460)
Supplement: Supplementary file 1 — Appendix S1 [file ALL-78-291-s001.docx]

**online supplementary material**

**Human neutrophils require short exposure to cytokines and allergen to become antigen-presenting cells**

Dominika Polak, PhD^1^, Adelheid Elbe-Bürger, PhD^2^, Claudia Kitzmüller, PhD^1^, Gerhard J. Zlabinger, MD^3^, and Barbara Bohle, PhD^1^

^1^Institute of Pathophysiology and Allergy Research, Center for Pathophysiology, Infectiology and Immunology, Medical University of Vienna, Vienna, Austria

^2^Department of Dermatology, Medical University of Vienna, Vienna, Austria

^3^Institute of Immunology, Medical University of Vienna, Vienna, Austria

**Corresponding author**

Barbara Bohle, PhD

Institute of Pathophysiology and Allergy Research, Center for Pathophysiology, Infectiology and Immunology, Medical University of Vienna, Austria,

Waehringer Guertel 18-20, 1090 Vienna, Austria

Phone: 0043-1-40400-5114, Fax: 0043-1-40400-6188

[barbara.bohle@meduniwien.ac.at](mailto:barbara.bohle@meduniwien.ac.at)

**Neutrophils** were isolated as described (1) from birch pollen-allergic individuals with rhinoconjunctivitis in spring, positive skin prick testing to birch pollen extract (ALK-ABELLÓ, Hørsholm, Denmark) and birch pollen-specific IgE of >0.35 kU_A_/L (ImmunoCAP; Thermo Fisher Scientific, Uppsala, Sweden) after written informed consent. The study was approved by the ethics committees of the Medical University of Vienna (EK 1344/2018) and conducted in accordance with the Declaration of Helsinki. The procedure resulted repeatedly in >99% pure CD16^+^CD66b^+^CCR3^−^HLA^-^DR^−^CD19^−^CD3^−^CD14^−^ neutrophils as determined by flow cytometry with a FACSCanto II by using FACSDiva (BD Biosciences, San Jose, Calif) and FlowJo (TreeStar, Ashland, Ore) software. Dead cells were stained with the Fixable Viability Dye eFluor 780 (eBioscience). The following anti-human antibodies were used: HLA-DR-allophycocyanin (APC), CD3-allophycocyanin-Cy7 (APC-Cy7), peridinin-chlorophyll-protein complex (PerCP)-labeled CD14 and CD19 (all from BD Bioscience); CD16-phycoerythrin (PE; clone 3G8), CD66b-Fluorescein isothiocyanate (FITC), CD123-PerCp, CCR3-allophycocyanin (APC) and CD63-PE (all from BioLegend, San Diego, Calif).

Neutrophils (1x10^6^/mL) were cultured in RPMI 1640 (Sigma Aldrich, Darmstadt, Germany) supplemented with 10% autologous plasma without or with GM-CSF (100 pg/mL), IFN-γ (10 ng/mL), and IL-3 (30 ng/mL; all from Peprotech, Rocky Hill, NJ).

Figure S1. **Cytokine exposure period and HLA-DR expression and viability of neutrophils**.


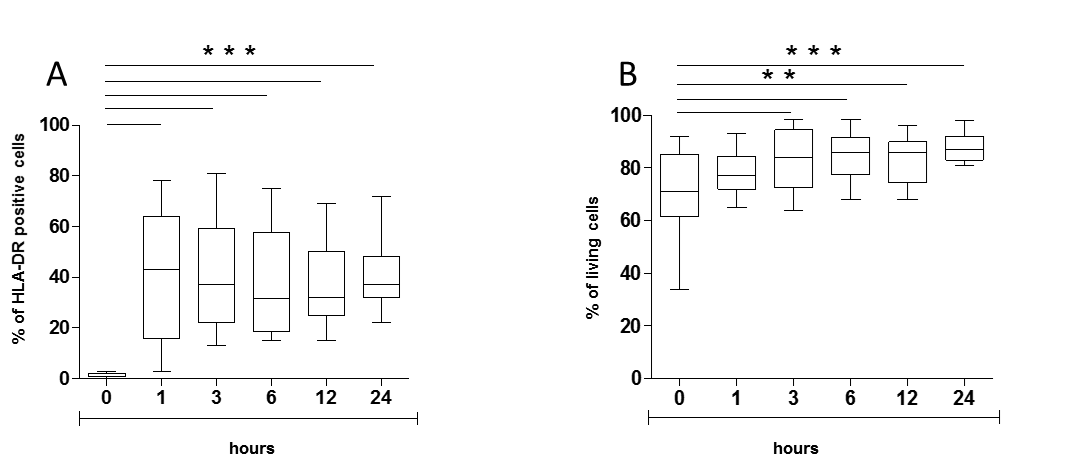


Neutrophils were stimulated with GM-CSF, IL-3, and IFN-γ for the indicated periods, washed, and maintained in medium. After 24h the percentages of HLA-DR-positive (A, n=5) and living (B) neutrophils were assessed by flow cytometry. **P<0.01, ***P<0.001, One-way ANOVA with Tukey's Multiple Comparison Test.

**Bet v 1-specific TCL** were expanded from PBMC from birch pollen-allergic individuals with recombinant Bet v 1.0101 (Bet v 1) as described (2). T-cells (2-5x10^4^) were incubated with irradiated (60 Gray) autologous neutrophils (5x10^4^) without and with Bet v 1 (5 µg/ml) or Bos d 5 (5 µg/ml, Sigma, Burlington, MA, USA). Proliferation was assessed as counts per minute (cpm) by addition of ^3^[H]-thymidine after 48h and incubation for additional 16h. All experiments were performed in duplicate. Cpm in cultures without allergen ranged from 194-17543 (median value 2026 cpm). Delta counts per minute (dpm) were calculated by subtracting cpm of medium controls from cpm of cultures stimulated with allergen. Stimulation indices (SI) were calculated as the ratio of cpm of stimulated cultures to cpm of unstimulated cultures.

**Human skin mast cells**

Full-thickness skin including subcutaneous fat was obtained from patients undergoing routinely performed body contouring surgeries. On clinical inspection and according to histology the skin was inconspicuous. Experiments were performed according to the Declaration of Helsinki principles after approval by the ethics committee of the Medical University of Vienna and written informed consent of the participants (EK 1149/2011). Subcutaneous fat and reticular dermis were removed and the remaining split thickness skin was cut into 0.5 cm² pieces and incubated in 2.4 U/ml dispase II (Roche, Vienna, Austria) overnight at 4°C. After separation of the epidermis, the dermis was digested in collagenase I (Gibco, Vienna, Austria) for 2 h at 37°C. CD117 mast cells were isolated using magnetic beads (MACS System, Miltenyi Biotec, Bergisch Gladbach, Germany) according to the manufacturer’s instructions. Cells were seeded in DMEM (Gibco) supplemented with 10% FCS, Penicillin/Streptomycin (both Biochrom, Berlin, Germany) and 100 ng/mL recombinant human stem cell factor (Peprotech, Rocky Hill, NY, USA). Mast cells were incubated with human IgE (13.6 µg/mL, Sigma Aldrich, Darmstadt, Germany) for 1 hour at 37˚C. Then, mast cells were either kept in medium or activated with an anti-human IgE antibody (13.6 µg/mL, Seracare Life Sciences Inc, Milford, Massachusetts, United States). Supernatants were collected after 24 hours and cytokine levels were assessed by using a bead array with the Luminex System 100 (Luminex, Austin, Tex).

Table S1**. Cytokines released by IgE-degranulated mast cells**

| **Donor*** | **Stimulus** | **Cytokines [ng/ml]** | | | | | | | |
| --- | --- | --- | --- | --- | --- | --- | --- | --- | --- |
|  |  | **GM-CSF** | **IFN-γ** | **IL-3** | **TNF-α** | **IL-4** | **IL-5** | **IL-6** | **IL-8** |
| **1** | Medium | 0.2 | <0.2 | 0.4 | <0.2 | <0.2 | <0.2 | 2.5 | 30.9 |
|  | αIgE | 53.1 | <0.2 | 0.4 | 47.8 | <0.2 | <0.2 | 2.5 | 44.4 |
| **2** | Medium | 1.4 | 0.3 | <0.2 | 1.0 | <0.2 | <0.2 | 12.1 | 283.7 |
|  | αIgE | 79.7 | <0.2 | 0.2 | 20.0 | <0.2 | <0.2 | 15.8 | 235.3 |
| **3** | Medium | 19.0 | <0.2 | <0.2 | 0.9 | <0.2 | 0.2 | 3.8 | 18.0 |
|  | αIgE | 78.9 | 0.3 | <0.2 | 37.2 | <0.2 | 0.2 | 6.4 | 19.5 |
| **4** | Medium | 2.7 | 0.5 | 5.1 | 4.2 | <0.2 | 0.2 | 29.8 | 1123 |
|  | αIgE | 957 | 0.8 | 12.1 | 64.2 | <0.2 | 1.5 | 14.8 | 1663 |
| **5** | Medium | 5.7 | 0.3 | 1.2 | 8.7 | <0.2 | <0.2 | 46.7 | 1700 |
|  | αIgE | 2826 | 1.4 | 36.4 | 173.3 | <0.2 | 2.3 | 27.7 | 2440 |

*from donors 1-3, 5x10^4^ cells/ml and from donors 4 and 5, 3x10^5^ cells/ml were used.

**Basophils** were enriched from PBMC to 53% of CCR3^+^CD123^+^ cells using the Basophil Isolation Kit II (Miltenyi Biotec. Bergisch Gladbach. Germany). Cells (1.5x10^5^/96 well) were cultured in RPMI 1640 (Sigma Aldrich) in the absence or presence of Bet v 1 (10 and 3 ng/ml). Supernatants were harvested after 48h and added to freshly isolated, autologous neutrophils.

Figure S2. **Mediators released from allergen-activated basophils induce HLA-DR expression on neutrophils**.


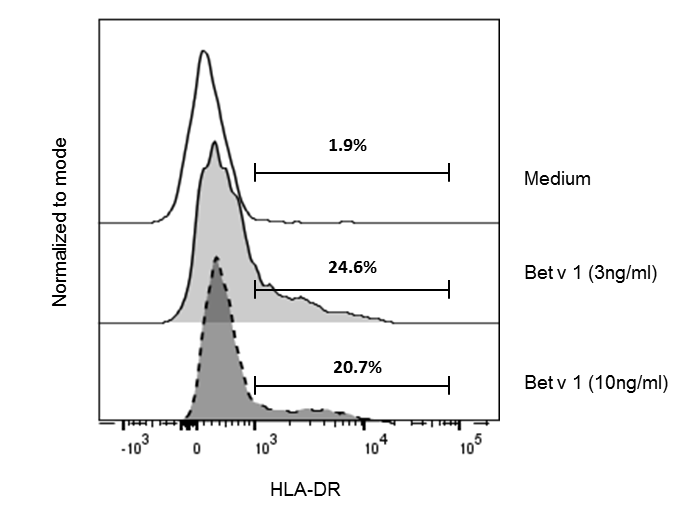


Supernatants from basophils cultured in the absence or presence of Bet v 1 were added to autologous neutrophils and HLA-DR expression was assessed after 24h by flow cytometry.

**References**

1. Polak D, Hafner C, Briza P, Kitzmuller C, Elbe-Burger A, Samadi N, et al. A novel role for neutrophils in IgE-mediated allergy: Evidence for antigen presentation in late-phase reactions. *J Allergy Clin Immunol* 2019;**143**(3):1143-1152 e1144.

2. Jahn-Schmid B, Radakovics A, Luttkopf D, Scheurer S, Vieths S, Ebner C, et al. Bet v 1142-156 is the dominant T-cell epitope of the major birch pollen allergen and important for cross-reactivity with Bet v 1-related food allergens. *J Allergy Clin Immunol* 2005;**116**(1):213-219.
